# Parent–child similarity on autism and ADHD traits and children's social functioning and psychological well‐being at 3 years

**DOI:** 10.1111/jcpp.70014

**Published:** 2025-07-11

**Authors:** Daniel L. Wechsler, Emily J. H. Jones, Greg Pasco, Tessel Bazelmans, Jannath Begum‐Ali, Mark H. Johnson, Tony Charman, The BASIS/STAARS Team, Mary Agyapong, Anna Blasi, Patrick Bolton, Celeste Cheung, Leila Dafner, Kim Davies, Mayada Elsabbagh, Mutluhan Ersoy, Janice Fernandes, Laurel Fish, Isobel Gammer, Teodora Gliga, Amy Goodwin, Jeanne Guiraud, Rianne Haartsen, Hanna Halkola, Alexandra Hendry, Rebecca Holman, Sarah Kalwarowsky, Anna Kolesnik, Michelle Liew, Sarah Lloyd‐Fox, Helen Maris, Luke Mason, Nisha Narvekar, Louise O'Hara, Andrew Pickles, Laura Pirazzoli, Helena Ribeiro, Erica Salomone, Chloë Taylor, Leslie Tucker

**Affiliations:** ^1^ Centre for Brain and Cognitive Development, Birkbeck University of London London UK; ^2^ Department of Psychology, Institute of Psychiatry, Psychology & Neuroscience King's College London London UK; ^3^ Department of Psychology University of Cambridge Cambridge UK

**Keywords:** Autism, ADHD, parent–child similarity, resilience, positive development

## Abstract

**Background:**

There is a pressing need for research on neurodevelopmental conditions to focus on predictors of resilient or positive outcomes, rather than core symptoms and impairment. One promising avenue is to consider whether child–parent similarity contributes to a protective family environment. For instance, investigations of the similarity–fit hypothesis have shown that parent–child attention‐deficit/hyperactivity disorder (ADHD) trait similarity is associated with more favourable parent or child ratings of parenting and parent–child interaction. However, very little similarity–fit research has focused on autism, and none to date has investigated whether parent–child trait similarity is more broadly predictive of children's outcomes beyond parent–child interaction. We assessed whether parent–child autism and ADHD trait similarity predicted children's social functioning and psychological well‐being in early childhood in a family history cohort.

**Methods:**

Our analytic sample comprised 222 children (45.5% female) and their parents from a longitudinal family history (autism and/or ADHD) cohort. A novel parent–child trait similarity measure was computed for autism and ADHD traits in each parent–child pair, and robust hierarchical regression was used to assess whether mother–child and father–child autism and ADHD similarity predicted children's social functioning and psychological well‐being at age 3 years, after accounting for the main effects of parent and child traits.

**Results:**

Mother–child autism trait similarity positively predicted both social functioning and psychological well‐being in children, while mother–child ADHD trait similarity positively predicted children's social functioning (but not well‐being). Furthermore, father–child autism trait similarity positively predicted children's social functioning, though it fell just short of statistical significance in outlier‐robust regression.

**Conclusions:**

Our findings suggest that parent–child neurodevelopmental trait similarity may act as a protective or promotive factor for children's early social functioning and psychological well‐being. Further work is warranted to determine whether there are similar effects in later childhood and to investigate the potential mechanisms underlying similarity–fit effects on children's outcomes.

## Introduction

Research on neurodevelopmental conditions such as autism and attention deficit/hyperactivity disorder (ADHD) has traditionally focused on the predictors, effects and underpinnings of core symptoms or traits, on relationships with co‐occurring conditions, or on various aspects of impairment (Appelqvist‐Schmidlechner, Lämsä, & Tuulio‐Henriksson, 2020; Sonuga‐Barke & Thapar, 2021). The shift towards a neurodiversity‐affirmative perspective on neurodevelopmental conditions has been accompanied by a broader (and largely separate) shift in psychiatric research, towards investigating predictors of resilience rather than vulnerability and pathology, and more recently, towards explicitly characterising positive development (Pellicano & den Houting, 2022; Pluess, 2024; Sonuga‐Barke, 2023). Resilience is increasingly recognised to be a multifaceted concept, relying not only on intrinsic factors (e.g., neurobiological factors) that may buffer the harmful effects of adverse exposures, but also on a dynamic process of coping with and adapting to challenges, and on protective factors in an individual's environment, such as the availability of social support (Ungar & Theron, 2020).

The family environment is a developmental context in which positive family interactions can scaffold the development of children's well‐being, functioning and overall resilience to challenges and adversities. Investigating protective or resilience factors in the family environment is particularly important in relation to neurodevelopmental conditions, which can present with various challenges for both children and their parents. For instance, parents of autistic children consistently report high levels of parenting stress (Hayes & Watson, 2013), and longitudinal family studies have indicated the presence of bidirectional relationships between parenting stress and mental health difficulties, and children's emotional and behavioural problems over time (Yorke et al., 2018; Zaidman‐Zait et al., 2014). Similarly, parents of children with ADHD report higher rates of parenting stress, family dysfunction, parent–child conflict and more self‐reported negative (and less positive) parenting behaviours (Claussen et al., 2024). At least some of these difficulties are attributable to ADHD traits in parents themselves, which have been shown to impact various aspects of parenting (Park & Johnston, 2019). However, as both autism and ADHD traits are highly heritable and often co‐occur in children and parents (Larsson, Chang, D'Onofrio, & Lichtenstein, 2014; Tung, Brammer, Li, & Lee, 2015), it is arguably important to consider not only the relative roles of parent and child traits, but also the ways in which these may interact to affect the parent–child relationship.

A modest but growing body of research has investigated whether parent–child similarity in neurodevelopmental traits influences the parent–child relationship (Johnston, Williamson, Noyes, Stewart, & Weiss, 2018). The similarity–fit hypothesis posits that neurodevelopmental trait similarity between parents and children should benefit the parent–child relationship, ostensibly by improving the behavioural compatibility or ‘fit’ of a parent–child dyad and thereby reducing conflict, improving understanding, or otherwise ensuring higher agreeability across interactions (Grimbos & Wiener, 2018). This hypothesis emerged from family ADHD research, but broadly mirrors the concept of double empathy in autism, which attributes some aspects of social and communication impairment experienced by autistic individuals to a mismatch in ‘neurotype’ between individuals, rather than to autism‐related deficits per se (Davis & Crompton, 2021; Milton, Gurbuz, & López, 2022). In empirical work, enhanced (or more effective) communication, understanding and rapport are reported both by participants and observers when two or more communicators are more closely matched on neurotype (Crompton, Ropar, et al., 2020; Crompton, Sharp, et al., 2020). However, very little research has explored the potential effects of autism trait similarity on interactions between parents and children.

Multiple similarity–fit studies have shown that parent–child similarity on ADHD traits can be beneficial for parenting and parent–child interaction. In a sample of 95 children (33% male, age ~ 5 to 10 years) and their mothers, Psychogiou, Daley, Thompson, and Sonuga‐Barke (2008) found that mothers in ‘mismatched’ parent–child pairs (i.e., mothers with low ADHD traits but whose children had high ADHD traits, and vice versa) reported less positive and involved parenting behaviour, as compared with mothers in ‘matched’ (i.e., high‐high and low‐low ADHD) pairs. In the same publication, authors reported a subsequent analysis of 192 preschool children (58% male), finding that mothers in mismatched ADHD pairs displayed less affectionate and constructive parenting behaviour than mothers in matched pairs. The lowest levels of affectionate and constructive parenting were observed in low‐ADHD mothers with high‐ADHD children. Similarly, in a sample of 156 children and early adolescents (100% male, age 5–13 years) and their parents, Johnston et al. (2018) found that more inattentive mothers, and more hyperactive–impulsive fathers, reported lower child‐directed empathy if their children did not meet criteria for ADHD (but not if children did meet criteria). Fathers with higher hyperactive–impulsive traits also reported lower encouragement of child autonomy only if children did not meet criteria for ADHD. Notably, in both of these studies, interactions between parent and child traits were seen in the absence of significant main effects of those traits, indicating that a neurodevelopmental trait mismatch – rather than parent or child ADHD traits per se – predicted less positive parenting behaviour.

Several studies have also supported the ‘similarity‐misfit’ hypothesis, wherein parent–child trait similarity (typically similarly high traits) may amplify associations between parent or child ADHD traits and adverse parenting or parent–child interactions. In a sample of 312 children (52% male, age ~ 5 to 10 years) and their parents, Psychogiou, Daley, Thompson, and Sonuga‐Barke (2007) assessed 278 mother and 85 father ratings of their own and their children's ADHD traits, and their negative parenting behaviour. Higher child ADHD traits predicted more negative parenting only in low‐ADHD mothers (indicating ameliorative or protective negative moderation by mother–child similarity), but in both low‐ADHD and especially high‐ADHD fathers (indicating amplificatory positive moderation by father–child similarity). A largely opposing pattern was reported in the only similarity–fit study to date, focusing on later adolescence. Grimbos and Wiener (2018) assessed mother and father ratings (156 each) of their own and their 156 adolescent children's (56% male, age 13–18 years) ADHD traits, and adolescent ratings of parent–child conflict. More inattentive adolescents reported more conflict with medium‐ and especially high‐inattentive mothers, but less conflict with low‐inattentive mothers. Conversely, more inattentive adolescents reported more conflict with medium‐ and especially low‐inattentive fathers, but slightly less conflict with high‐inattentive fathers. These findings suggest that even when parents rate their own and children's ADHD traits, any potential trait similarity effects can differ by parent, by child age and/or by outcome measure (including whether parents or children report the outcome).

Only one study to date has investigated the similarity–fit hypothesis with respect to autism traits. In a sample of 143 children (100% male, age 6–11 years) and their mothers, Ward (2022) found that mothers with higher autism traits reported less positive parenting behaviour and higher parenting stress, with no main effects of child autism nor moderation by mother–child autism similarity. Consistent with previous findings, mother–child ADHD similarity negatively moderated (i.e., ameliorated) associations between both maternal and child ADHD traits and mother‐reported negative parenting behaviour, as well as parenting stress. The highest levels of parenting stress were reported by low‐ADHD mothers with high‐ADHD children, whereas high‐ADHD mothers reported roughly equal (moderate) levels of parenting stress regardless of child ADHD traits. Interestingly, mothers in matched high‐ADHD pairs reported some of the lowest levels of negative parenting behaviour, comparable to those in matched low‐ADHD pairs.

Along with the sparsity of autism‐focused work, a more fundamental gap in existing similarity–fit studies has been the focus on specific parental behaviours or dyadic measures of parent–child interaction. While these are a proximal and therefore natural focus of study on the potential benefits of parent–child neurodevelopmental similarity, an unexplored yet relevant question is whether parent–child neurodevelopmental trait similarity exerts a broader effect on children's development, for instance, on functional or mental health outcomes. The similarity–fit concept is well‐placed for investigating broader effects on development. Whereas the effect of any individual feature of parenting on a child's development is difficult to establish empirically, neurodevelopmental trait similarity between parents and children may affect a range of behaviours over time, conferring cumulative benefits or harms to the parent–child relationship and children's wider rearing environment, which in turn shape a variety of child developmental outcomes including those commonly co‐occurring with neurodevelopmental conditions (Crowell, Keluskar, & Gorecki, 2019; Deault, 2010). As parental neurodevelopmental traits will have manifested and should remain largely stable across their children's lives (Karam et al., 2015; Yarar et al., 2022), any cumulative effects of parent–child similarity, while depending partly on the onset and presentation of neurodevelopmental traits in children, can be expected to manifest early and to remain observable throughout development.

We set out to build on and expand the extant similarity–fit literature while addressing several gaps and limitations. Namely, we aimed to investigate autism as well as ADHD trait similarity–fit effects, for both mothers and fathers, in a roughly equal sample of boys and girls recruited in infancy as part of the BASIS/STAARS family history cohort. This cohort is enriched for neurodevelopmental traits, as most children have a first‐degree relative with a diagnosis of autism and/or ADHD. We assessed whether parent–child similarity predicted children's social functioning and psychological well‐being, both being indicators of resilient or otherwise positive development in the context of the challenges faced by neurodivergent individuals (Black et al., 2024). Finally, we noted limitations with the previously adopted approach of operationalising similarity–fit effects using a conventional interaction term between standardised parent and child scores. To more accurately represent phenotypic similarity or ‘fit’ between parents and children, we constructed a novel similarity–fit measure based on the absolute difference between parent and child standardised scores.

## Methods

### Sample

The BASIS/STAARS study is a longitudinal, prospectively enrolled infant‐sibling cohort of children with elevated likelihood for autism and/or ADHD (Begum Ali, Charman, Johnson, Jones, & BASIS/STAARS Team, 2020; Bazelmans et al., 2024). Elevated likelihood infant siblings were recruited at 5 or 10 months of age by virtue of having a first‐degree relative (an older sibling and/or parent) with a diagnosis of autism and/or ADHD (or elevated ADHD traits) at birth (see Tables S1 and S2). A smaller group of comparison infants with a typically developing older sibling and no family history of autism or ADHD were also recruited. Other inclusion criteria were full‐term birth (gestational period of at least 36 weeks) and no known medical or developmental condition. For full recruitment and ascertainment details, see Elsabbagh et al. (2013) and Charman et al. (2023). The analytic sample (*N* = 222, 101 female; 45.5%) comprised 111 infants with a family history of autism, 17 with a family history of ADHD, 50 with a family history of autism and ADHD and 44 with no family history. Table 1 displays sample demographic information by family history group.

**Table 1:** Demographic characteristics by family history sampling frame

Family history group	Autism *N* = 111	Autism + ADHD *N* = 50	ADHD *N* = 17	TL *N* = 44
Sex (child)				
Male (%)	57 (51%)	28 (56%)	11 (65%)	25 (57%)
Female (%)	54 (49%)	22 (44%)	6 (35%)	19 (43%)
Age in months (child)				
Mean (*SD*)	38.19 (1.87)	38.52 (1.99)	37.03 (1.27)	37.95 (1.70)
Ethnicity (mother)				
White/European/Irish (%)	95 (86%)	48 (96%)	16 (94%)	39 (89%)
Asian/African/African–Caribbean/Mixed heritage (%)	16 (14%)	2 (4%)	1 (6%)	5 (11%)
Highest level of education (mother)				
Up to high school/Further education (%)	35 (32%)	20 (41%)	4 (24%)	3 (7%)
University degree or higher (%)	76 (68%)	29 (59%)	13 (76%)	40 (93%)

Maternal education data were not available for two families. TL, typical likelihood (no family history of autism nor ADHD).

### Measures and linkage

All child trait measures were reported by primary caregivers (typically mothers) at 36 months. Child autism and ADHD traits were assessed using the Social Responsiveness Scale, Second Edition (SRS; Constantino & Gruber, 2012) and the Child Behaviour Checklist 18 m‐5y (CBCL; Achenbach & Rescorla, 2000) DSM Attention Deficit/Hyperactivity Problems scale, respectively. Child social functioning was assessed using the Vineland 2 Adaptive Behaviour Scale (VABS; Sparrow, Balla, & Cicchetti, 2005) Socialisation Dimension score (consisting of the Interpersonal Relationships, Play and Leisure and Coping Skills subdimensions). Child psychological well‐being was assessed using a reversed mean composite of children's standardised scores on the CBCL DSM Affective Problems and Anxiety Problems scales.

Parent trait measures were self‐reported by mothers and fathers when children were aged 24 to 36 months, and/or when children were aged 6–12 years.^1^ Parent autism and ADHD traits were assessed using the SRS Adult Self‐report Form (Constantino & Gruber, 2012) and the Conners Adult ADHD Rating Scales Self‐report: Long Version (CAARS; Conners, Erhardt, & Sparrow, 2002), respectively.

Data linkage was carried out to derive parent–child pairings based on available data, linking child autism/ADHD measures at 36 months with the closest available parent measure. In most cases (59% across paired samples), ‘ideal’ pairs of child and parent measures, both collected in early childhood (roughly a year apart), were attained. Specifically, matching early childhood measures were available in 53.2% and 57.6% of mother‐ and father–child autism pairs, respectively, and in 60.4% and 64.6% of mother‐ and father–child ADHD pairs, respectively. In the remaining pairs, child measures collected in early childhood were linked with parent measures collected during mid‐childhood visits (typically two to six years apart).

Mother‐ and father–child similarity measures were constructed for autism and for ADHD traits by computing the absolute difference between standardised parent and child scores and reversing difference scores to derive an absolute similarity measure, such that the most dissimilar pair in each sample was anchored at the minimum absolute similarity score of zero, with higher similarity scores corresponding proportionally to lower trait disparity between parents and children (see Appendix S1 for rationale and computation steps).

Standard covariates included child sex and child and parent age. In addition, a parent–child measure interval covariate was computed to account for the potential confounding effect of the variable lag in days between child and parent measure collection.

We used a data‐driven approach to apply Box‐Cox power transformations (Atkinson, Riani, & Corbellini, 2021) to primary predictors and outcome measures as appropriate (see Appendix S2 for details), prioritising transformations which: (a) improved uniformity of bivariate predictor‐outcome correlations while retaining the correlational structure between variables; (b) reduced non‐normality of error terms while also producing consistent SEs when estimated with HR‐OLS.

### Analyses

As in previous studies, hierarchical multiple regression was used to assess whether parent–child similarity predicted child outcomes after controlling for the main effects of child and parent traits and any covariates. For each outcome and each parent–child autism/ADHD pairing, an initial main effects model regressed the outcome on all main predictors and covariates, while a subsequent full model included parent–child similarity as an additional predictor. ANOVA comparisons were then used to assess whether there was a significant increase in variance in outcome explained by the Full model.

A triangulation approach was used to compare results from ordinary least squares (OLS) regressions with those derived using several robust methods. Heteroscedasticity‐robust standard errors (SEs) were computed for OLS parameter estimates using the HC3 covariance matrix, the advised heteroscedasticity‐consistent matrix for samples of 250 or less (Long & Ervin, 2000). In addition, outlier‐robust parameter estimates were computed using the MM‐estimator (Koller & Stahel, 2017; Yohai, 1987), which downweights the otherwise disproportionate influence of outliers on parameter estimates. We considered findings to be robust when they were consistent across OLS, OLS with heteroscedasticity‐robust SEs (HR‐OLS), and MM‐estimator (MM) regressions.

## Results

Table 2 displays descriptive statistics for all parent and child measures in the combined analytic sample, which included 222 children with corresponding parent autism and/or ADHD data. Table S3 displays correlations between all phenotypic measures and main covariates.

**Table 2:** Descriptive statistics of raw variables across combined analytic sample

	*N*	Mean	*SD*	Median	Min.	Max.	Skew.	Kurt.
*Child measures*								
Child Vineland socialisation standardised score	195	97.56	12.43	100	61	124	−0.89	0.51
Child CBCL DSM affective problems total	189	2.55	2.99	2	0	13	1.49	1.72
Child CBCL DSM anxiety problems total	189	3.01	3.08	2	0	14	1.27	1.28
Child SRS total score	212	35.67	28.41	27	3	142	1.77	2.55
Child CBCL DSM ADHD total	189	4.51	3.29	4	0	12.	0.58	−0.75
Child Age (months)	222	38.13	1.85	37.80	33.77	49.38	1.48	5.48
*Mother measures*								
Mother SRS Total	205	35.20	27.10	27	1	124	1.41	1.48
Mother CAARS DSM ADHD symptoms total	182	13.78	10.09	11	1	49	1.18	1.09
Mother Age (years)	220	38.62	4.30	38.74	21.30	49.25	−0.38	0.60
*Father measures*								
Father SRS Total	177	44.91	28.85	37	2	130	0.77	−0.11
Father CAARS DSM ADHD symptoms total	161	14.56	10.29	12	0	52	0.98	1.11
Father Age (years)	205	40.80	4.99	40.64	23.82	56.19	0.17	0.74
*Collection interval covariates*								
Mother–child autism collection interval (months)	205	36.22	29.81	18	0	105	0.67	−1.16
Father–child autism collection interval (months)	177	35.43	30.99	13	0	105	0.77	−1.10
Mother–child ADHD collection interval (months)	182	29.95	26.56	13	0	96	1.16	−0.08
Father–child ADHD collection interval (months)	161	29.83	27.98	13	0	96	1.22	−0.08

CAARS, Conners Adult ADHD Rating Scales; CBCL, Child Behaviour Checklist; SRS, Social Responsiveness Scale.

Tables 3 and 4 display model statistics and standardised parameter estimates from OLS and MM regressions, respectively (for covariate results, see Appendix S3, Tables S4 and S5). While most substantive findings were consistent between methods, some findings from OLS and HR‐OLS regressions were not replicated in MM regressions. Estimates and plots are hereafter reported for full OLS regressions, while noting any relevant differences seen in MM regressions. Broadly, full autism trait models explained a comparable proportion of variance in social functioning (39%–45%) and psychological well‐being (46%–48%), while Full ADHD trait models explained substantially less variance in social functioning (15%–16%) than in psychological well‐being (34%–38%).

**Table 3:** Model summaries for OLS regressions, including standardised parameter estimates for main effects of child and parent autism/ADHD, and effects of parent–child similarity

Similarity phenotype	Child outcome	*N*	Model	β (*p*)			Adj. *R* ^2^
				Child	Parent	Similarity	
Mother–child Autism	Social functioning	179	1	−.56 (<.001)***	.02 (.770)	–	0.362
		179	2	−.54 (<.001)***	.06 (.361)	.18 (.005)**	**0.387**
	Psychological well‐being	172	1	−.59 (<.001)***	−.07 (.291)	–	0.431
		172	2	−.55 (<.001)***	−.04 (.561)	.20 (.001)**	**0.463**
Mother–child ADHD	Social functioning	158	1	−.32 (<.001)***	−.12 (.123)	–	0.134
		158	2	−.30 (<.001)***	−.10 (.208)	.17 (.030)*	**0.155**
	Psychological well‐being	182	1	−.53 (<.001)***	−.16 (.009)**	–	0.376
		182	2	−.52 (<.001)***	−.15 (.015)*	.09 (.120)	0.381
Father–child Autism	Social functioning	155	1	−.58 (<.001)***	−.12 (.077)	–	0.439
		155	2	−.54 (<.001)***	−.09 (.187)	.14 (.034)*	**0.452**
	Psychological well‐being	151	1	−.61 (<.001)***	−.18 (.006)**	–	0.482
		151	2	−.61 (<.001)***	−.18 (.007)**	−.01 (.915)	0.478
Father–child ADHD	Social functioning	134	1	−.38 (<.001)***	−.06 (.465)	–	0.156
		134	2	−.38 (<.001)***	−.06 (.479)	<.01 (.961)	0.149
	Psychological well‐being	155	1	−.51 (<.001)***	−.17 (.020)*	–	0.347
		155	2	−.51 (<.001)***	−.17 (.023)*	−.01 (.876)	0.343

***p < .001; **p < .01; *p < .05.

**Table 4:** Model summaries for MM regressions, including standardised parameter estimates for main effects of child and parent autism/ADHD, and effects of parent–child similarity

Similarity phenotype	Child outcome	*N*	Model	β (*p*)			Adj. *R* ^2^
				Child	Parent	Similarity	
Mother–child Autism	Social functioning	179	1	−.52 (<.001)***	−.01 (.840)	–	0.340
		179	2	−.52 (<.001)***	.06 (.407)	.19 (.003)**	**0.383**
	Psychological well‐being	172	1	−.56 (<.001)***	−.12 (.085)	–	0.435
		172	2	−.55 (<.001)***	−.04 (.529)	.22 (<.001)***	**0.465**
Mother–child ADHD	Social functioning	158	1	−.30 (<.001)***	−.14 (.080)	–	0.134
		158	2	−.29 (<.001)***	−.11 (.175)	.17 (.037)*	**0.155**
	Psychological well‐being	182	1	−.52 (<.001)***	−.21 (<.001)***	–	0.397
		182	2	−.52 (<.001)***	−.19 (.002)**	.07 (.240)	0.398
Father–child Autism	Social functioning	155	1	−.59 (<.001)***	−.12 (.074)	–	0.450
		155	2	−.56 (<.001)***	−.09 (.189)	.13 (.053)	0.460
	Psychological well‐being	151	1	−.64 (<.001)***	−.20 (.002)**	–	0.507
		151	2	−.64 (<.001)***	−.21 (.002)**	−.02 (.778)	0.503
Father–child ADHD	Social functioning	134	1	−.38 (<.001)***	−.09 (.321)	–	0.154
		134	2	−.38 (<.001)***	−.09 (.362)	.01 (.922)	0.147
	Psychological well‐being	155	1	−.52 (<.001)***	−.16 (.030)*	–	0.334
		155	2	−.52 (<.001)***	−.16 (.037)*	−.01 (.944)	0.329

***p < .001; **p < .01; *p < .05.

**Figure 1:**
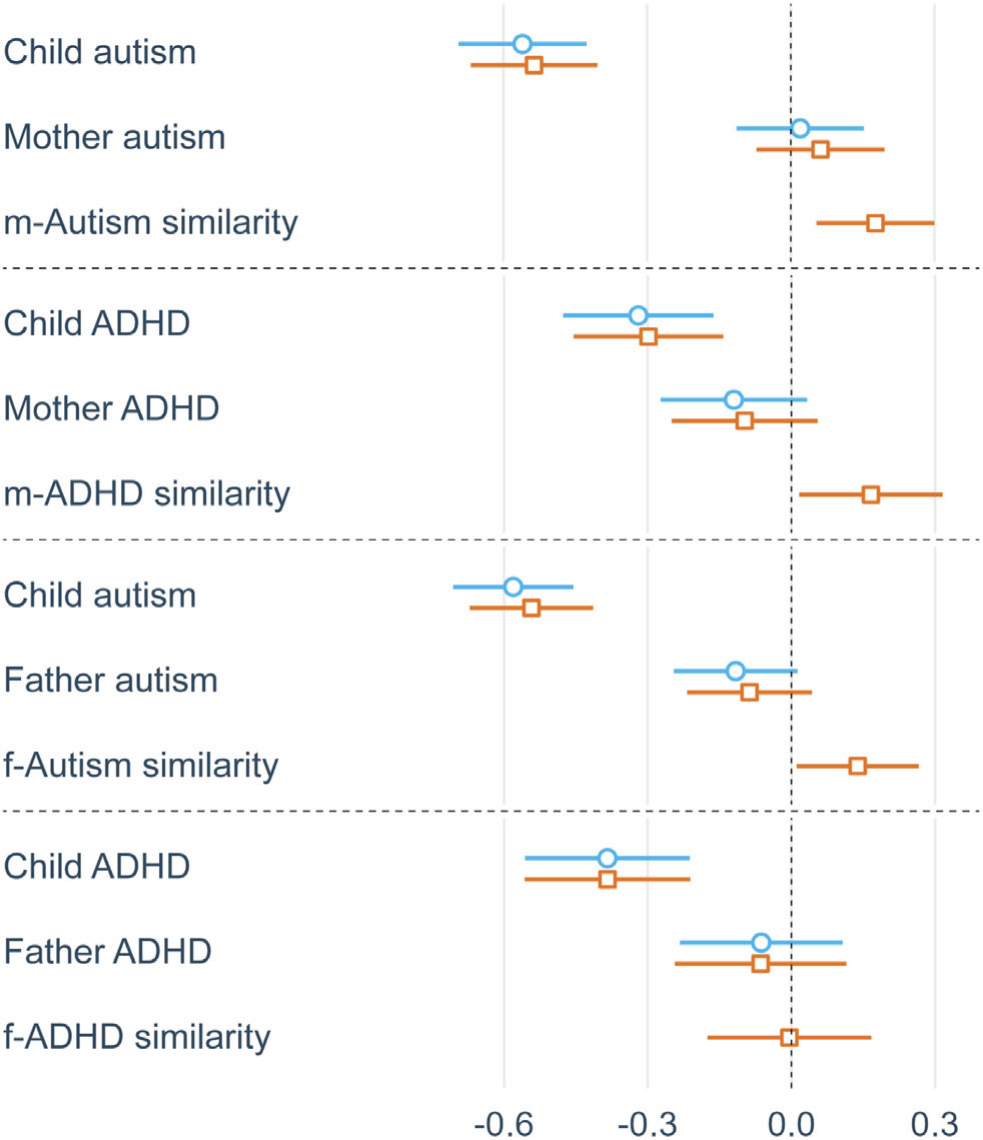
Summary plots from main effects (blue) and full (orange) social functioning OLS regressions, displaying standardised parameter estimates of main parent and child effects and parent–child similarity effects

**Figure 2:**
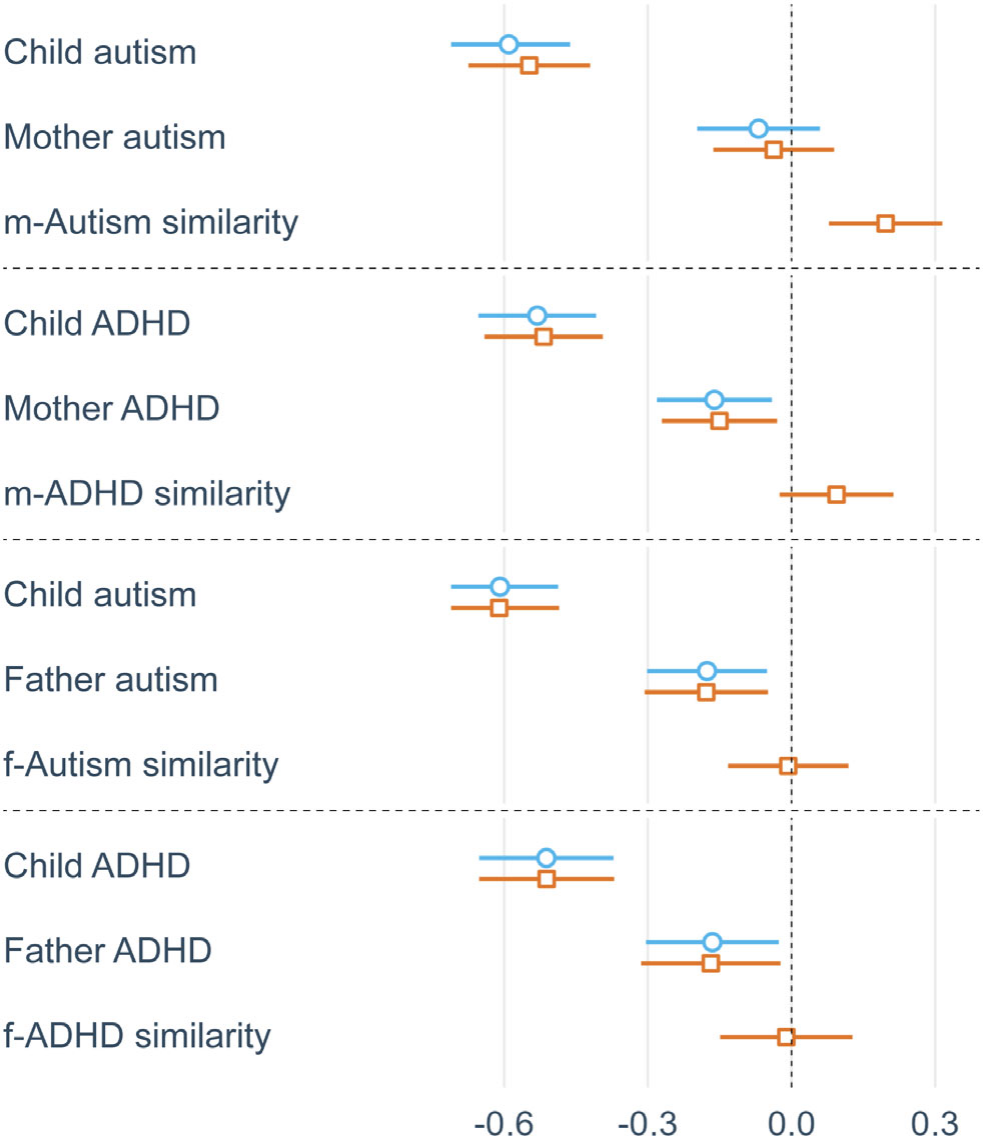
Summary plots from main effects (blue) and full (orange) psychological well‐being OLS regressions, displaying standardised parameter estimates of main parent and child effects and parent–child similarity effects

Figures 1 and 2 display summary plots for social functioning and psychological well‐being analyses, respectively, showing standardised parameter estimates and confidence intervals from main effects and full models. Child autism and ADHD traits consistently and negatively predicted social functioning (β = −.54 to −.30; all *p* < .001) and psychological well‐being (β = −.61 to −.51; all *p* < .001). Parent traits did not predict children's social functioning, but maternal ADHD (β = −.15; *p* = .015), paternal ADHD (β = −.17; *p* = .023) and paternal autism (β = −.18; *p* = .007) traits negatively predicted children's psychological well‐being.

Mother–child autism similarity positively predicted children's social functioning (β = .18; *p* = .005) and psychological well‐being (β = .20; *p* = .001), while mother–child ADHD similarity positively predicted children's social functioning (β = .17; *p* = .030) but not well‐being. Father–child autism trait similarity positively predicted children's social functioning in OLS regressions (β = .14; *p* = .034), but fell just below statistical significance in MM regressions (β = .13; *p* = .053). Notably, the magnitude of all significant similarity–fit effects was substantial, with effect sizes at 37%–57% the magnitude of the negative main effects of respective child traits.

## Discussion

We used a novel and analytically robust approach to investigate the similarity–fit hypothesis in a prospectively enrolled autism and ADHD family history sample. Whereas existing similarity–fit research has focused primarily on ADHD and on specific aspects of parenting and parent–child interaction, we assessed whether parent–child autism and ADHD trait similarity predicted children's social functioning and psychological well‐being at 3 years. Broadly consistent with previous work, we found that neurodevelopmental trait similarity predicted more favourable outcomes after accounting for the main effects of parent and child traits. Specifically, autism trait similarity with mothers predicted higher social functioning and psychological well‐being, while ADHD trait similarity with mothers predicted higher social functioning. Autism trait similarity with fathers also predicted higher social functioning, though it fell just short of statistical significance in robust regressions. While this indicates that extreme scores on predictors drove some portion of the estimated effect, it is arguably unwise to dismiss this finding outright, particularly given the smaller sample available for father–child analyses. In sum, our findings situate parent–child neurodevelopmental similarity as a positive influence on the development of social functioning and psychological well‐being, the benefits of which are evident by early childhood. Most notably, ours is the first study to find evidence for the benefits of autism trait similarity, which emerged as a more extensive predictor of child outcomes in our analyses than did ADHD trait similarity. The only other study to investigate autism similarity–fit effects found that autism trait similarity did not predict positive or negative parenting, nor parenting stress, in mothers of boys aged 6–11 years, nearly half of whom were diagnosed with autism (Ward, 2022). Our findings may indicate that autism trait similarity plays a more important role in early childhood than in later childhood, or could reflect our decision to focus on children's early outcomes rather than on specific aspects of parenting. Future research could investigate this by assessing whether parent–child autism trait similarity predicts children's outcomes in later years.

Given recent discussion of the value of studying positive development, rather than resilience in the context of psychopathology (Pluess, 2024), an important question is whether our findings position parent–child similarity as a promotive or broadly positive developmental factor, rather than a protective or resilience factor against the ostensibly harmful effects of child and/or parent neurodevelopmental traits. It is notable that prior similarity–fit research provided very little evidence for moderation of main effects of parent or child traits by parent–child similarity (Johnston et al., 2018). Instead, parent–child similarity effects (operationalised as interaction terms) were typically seen in the absence of main effects of parent or child traits. As we used pre‐computed absolute similarity measures rather than assessing moderation using interaction terms, any effects of parent–child similarity on the outcomes in our study were independent of (i.e., controlling for) any effects of child and parent traits. Similarity–fit effects in our analyses were not negligible in magnitude and acted in the opposing direction of negative child trait effects. Therefore, a properly contextualised interpretation of our results could be that parent–child similarity acts as a promotive or broadly positive factor in the context of a sample where most children are at elevated likelihood of developing autism and ADHD.

Previous similarity–fit hypothesis findings, that parent–child similarity improves various aspects of parenting, parenting stress and parent–child interaction (Grimbos & Wiener, 2018; Johnston et al., 2018; Ward, 2022), are likely to be relevant to our findings regarding psychological well‐being, given evidence that parenting stress and negative parenting behaviour prospectively predict internalising problems in children with autism and/or ADHD (Claussen et al., 2024; Zaidman‐Zait et al., 2014). While it is less clear how parent–child similarity might lead to improved social functioning in children, these findings could be informed by the Double Empathy concept, which emphasises benefits to social communication between individuals who are more neurodevelopmentally similar (Milton et al., 2022). Parents play a predominant role in children's early development, including for many children the majority of interpersonal contact in the early years, as well as the facilitation of social activities outside the home. As such, parents who are more neurodevelopmentally similar to their children may benefit from a better understanding of their children's strengths and challenges, and so may be better able to support them in finding ways to socially engage and develop healthy social relationships early in life. This could include seeking out other parents of neurodivergent children to facilitate social development with ‘better‐matched’ others, in environments that are less likely to be stigmatising of differences associated with autism and ADHD traits (Accardo, Neely, Pontes, & Pontes, 2024). It could also include ensuring children have access to sensory or other accommodations, and more specialised supervision, during their early social interactions.

One issue that may bear on the implications of our findings is the role of genetic and/or environmental confounding on relationships between parent traits and child outcomes, and in particular the extent to which the protective effects of parent–child similarity could be explained by unobserved influences on neurodevelopmental trait covariance within parent–child dyads. While our analyses did account for the main effects of child and parent autism and ADHD traits, there may still be co‐occurring developmental features that were not adequately controlled for by core trait measures, but which independently affected both children's outcomes and parent–child trait similarity. For example, given the high heritability of both autism and ADHD traits (Larsson et al., 2014; Tick, Bolton, Happé, Rutter, & Rijsdijk, 2016), greater intra‐pair similarity should generally indicate a greater influence of common genetic variation on neurodevelopmental traits in the affected dyad. In turn, greater dissimilarity could indicate a more predominant role of copy number variations (CNVs) or other de novo genetic variants on the higher‐scoring individual's trait variation. These variants are associated with greater impairment and deleterious effects on health (Plomin, DeFries, Knopik, & Neiderheiser, 2013), the unobserved impacts of which could have a confounding effect on associations between parent–child similarity and children's outcomes. This could be tested in future work by incorporating parent and child polygenic scores to control for trait similarity attributable to common variation. If this resulted in an increase in the magnitude of similarity–fit effects, this would indicate that some portion of the poorer outcomes seen in dissimilar pairs was attributable to uncontrolled confounding by correlates of rarer genetic variants.

### Strengths and limitations

The current work had multiple methodological strengths, using a robust analytic methodology to investigate a relatively understudied putative protective or promotive factor for children's early outcomes in the context of resilience and positive development. Furthermore, we constructed a novel measure of parent–child similarity that consistently captured the distance between each parent's and child's position on their standard score distribution, without weighting scores based on whether parents or children were high, low, or average scorers. This measure is intuitive to compute and can be adopted in future research on similarity–fit effects of various phenotypes, in parent–child as well as sibling dyads, and on a range of outcomes.

Several limitations were also present. First, primary caregivers (mostly mothers) rated both their own and their children's autism and ADHD traits, as well as children's psychological well‐being, potentially introducing bias due to shared rater and method variance. While mothers typically rated their own autism and ADHD traits at least a year apart from child ratings (with ~40% of ratings being several years apart), some of the observed similarity–fit effects could nonetheless partly reflect primary caregivers' perceived similarities or differences with their children. Separately, parents' actual neurodevelopmental similarity with children could affect their perceptions of children's functioning and well‐being. Additionally, as mothers were primary caregivers in the majority of our sample, mother‐specific effects could also reflect increased daily contact and familiarity with children. Future work could investigate these possibilities by using partner or clinician reports of parent and/or child traits, child ratings of their own traits and more families where fathers are primary caregivers.

Second, the onset of substantive ADHD traits is likely to occur later than age 3 years in many children in our sample (Egger, Kondo, & Angold, 2006; Rocco, Corso, Bonati, & Minicuci, 2021). While the main effects of child ADHD traits were consistently observed, a lack of fully manifest ADHD traits in a substantial portion of children may explain our relative lack of findings with regard to parent–child ADHD similarity. Future studies examining later outcomes could be more informative as to ADHD trait similarity–fit effects.

Third, while we set out to investigate parent–child similarity as a protective or positive developmental factor rather than following a conventional focus on impairment and dysfunction, it should be noted that the Vineland and reversed CBCL scales used to assess social functioning and psychological well‐being were conceived in clinical contexts. As such, dysfunction‐based biases will have affected what these measures captured. There remains an unmet need for research explicitly assessing and predicting positive developmental outcomes (Pluess, 2024). This is particularly true for those with neurodevelopmental conditions, given the overwhelming historical research focus on dysfunction in relation to these conditions, and interventions largely aiming for normative developmental outcomes as defined by others.

Fourth, our use of a volunteer family history sample meant that findings may not be generalisable to other populations, for instance, in children who are clinically diagnosed (and who may not have a diagnosed first‐degree relative), or in families from a lower socioeconomic background. As most families had cohabiting biological parents, findings may also not generalise to extended family structures. Replication of our findings in different samples is warranted to establish whether parent–child similarity is more or less important in specific groups. For instance, it could be less beneficial in families where parents and/or children have particularly elevated traits, or more beneficial in the context of the reduced access to clinical and support services experienced by those of lower socioeconomic backgrounds.

With access to larger samples, future researchers could jointly assess the relative effects of mother‐ and father–child similarity, and the relative effects of autism and ADHD trait similarity. This is important because autism and ADHD traits share substantial phenotypic overlap (Taylor et al., 2013), but have varying phenotypic and aetiological relationships with different traits that may mediate similarity–fit effects (Ghirardi et al., 2021; Tistarelli, Fagnani, Troianiello, Stazi, & Adriani, 2020). In addition, the effects of neurodevelopmental trait similarity with one parent are not likely to act independently of similarity with other family members (i.e., the other parent and any siblings). Family‐wide and multi‐trait analyses are an important future step for exploring both the relative importance of autism and ADHD trait similarity, and the relative importance of similarity–fit effects with different family members. Future work could also investigate subtype‐specific effects, since girls (and their mothers) are likely to experience more inattentive and less prominent hyperactive–impulsive traits (Hinshaw, Nguyen, O'Grady, & Rosenthal, 2022), and less pronounced social and/or communication difficulties (Wood‐Downie, Wong, Kovshoff, Cortese, & Hadwin, 2021), as opposed to boys (and their fathers). Previous studies have indicated the presence of parent‐ and subtype‐specific similarity–fit effects in relation to ADHD and positive parenting behaviours (Johnston et al., 2018). Similar distinctions may be worth exploring in relation to parent‐ or dyad‐specific similarity–fit effects on child outcomes.

## Conclusions

We present evidence that parent–child similarity in autism and ADHD traits may act as a protective or broader positive influence on children's early development of social functioning and psychological well‐being. Several aspects of our investigation were novel, including assessing similarity–fit via an intuitively computed absolute similarity variable that is easily replicated. Future studies wishing to adopt this method could investigate a wider range of developmental outcomes across development. Larger samples would also allow for family‐wide analyses and those jointly assessing the roles of autism or ADHD trait similarity. Separately, understanding the mechanisms underpinning any observed similarity–fit effects is important for translation into interventions. For instance, future studies could test whether certain parent‐mediated interventions are better suited to neurodevelopmentally dissimilar parent–child pairs (Roberts et al., 2023). Finally, there remains a need for research focusing explicitly on defining and understanding positive developmental outcomes in neurodivergent populations.

## Ethical information

Ethical approval was obtained from the NHS National Research Ethics Service (NHS RES London REC 14/LO/0170, 13/LO/0751, 08/H0718/76 and 06/MRE02/73), the Psychiatry, Nursing and Midwifery Research Ethics Subcommittee, King's College London (RESCM‐18/19‐10,556) and the Research Ethics Committee, Department of Psychological Sciences, Birkbeck, University of London. Parents provided written informed consent.

**Key points**

**What's known**

- Multiple studies have found that parent–child ADHD trait similarity predicts more positive and less negative parenting behaviour, less parenting stress and less parent–child conflict. No studies have found similar benefits of autism trait similarity.

**What's new**

- We provide the first evidence that autism and ADHD trait similarity between parents and children are protective or promotive factors for child development, predicting higher social functioning and/or psychological well‐being in early childhood.

**What's relevant**

- Future research could explore whether parent–child neurodevelopmental similarity predicts a wider range of outcomes, and at various ages. Understanding the mechanisms underpinning these benefits is also an important area of study, if findings are to be used to inform interventions.

## Supporting information


**Table S1.** Autism family history ascertainment by family history group.
**Table S2.** ADHD family history ascertainment by family history group.
**Table S3.** Pearson's *r* correlations between measures across combined analytic sample.
**Table S4.** Standardised parameter estimates of covariate effects from OLS regressions.
**Table S5.** Standardised parameter estimates of covariate effects from MM regressions.
**Figure S1.** Plots comparing interaction terms with absolute similarity measures.
**Appendix S1.** Parent‐child similarity measure.
**Appendix S2.** Transformations.
**Appendix S3.** Covariate effects.

## Data Availability

Data available following a review of requests as indicated here: https://www.basisnetwork.org/collaboration‐and‐project‐affiliation/index.html.

## Endnote

1 For a sub‐sample of the participants followed‐up after the 3‐year assessment.
