# Supplementary material for: Parent–child similarity on autism and ADHD traits and children's social functioning and psychological well‐being at 3 years
Source: J Child Psychol Psychiatry. 2025 Jul 11;66(12):1818–28. doi: 10.1111/jcpp.70014 (PMC12626176; doi:10.1111/jcpp.70014)
Supplement: Supplementary file 1 — Table S1. Autism family history ascertainment by family history group. Table S2. ADHD family history ascertainment by family history group. Table S3. Pearson's r correlations between measures across combined analytic sample. Table S4. Standardised parameter estimates of covariate effects from OLS regressions. Table S5. Standardised parameter estimates of covariate effects from MM regressions. Figure S1. Plots comparing interaction terms with absolute similarity measures. Appendix S1. Parent‐child similarity measure. Appendix S2. Transformations. Appendix S3. Covariate effects. [file JCPP-66-1818-s001.docx]

Parent-Child Similarity on Autism and ADHD Traits and Children’s Social Functioning and Psychological Wellbeing at 3 Years

# Supplementary Materials

| Table S1. Autism family history ascertainment by family history group. | | | | | | |
| --- | --- | --- | --- | --- | --- | --- |
| Family history group | Typical likelihood | Autism | Autism + ADHD | ADHD | Total | Percent |
| Not elevated likelihood for autism | 44 | - | - | 17 | 61 | 27.48% |
| Sibling diagnosed with autism | - | 104 | 44 | - | 148 | 66.67% |
| Parent diagnosed with autism | - | 0 | 1 | - | 1 | 0.45% |
| Sibling and parent diagnosed with autism | - | 6 | 5 | - | 11 | 4.95% |
| Half-sibling diagnosed with autism | - | 1 | 0 | - | 1 | 0.45% |
| Total | 44 | 111 | 50 | 17 | 222 |  |

| Table S2. ADHD family history ascertainment by family history group. | | | | | | |
| --- | --- | --- | --- | --- | --- | --- |
| Family history group | Typical likelihood | Autism | Autism + ADHD | ADHD | Total | Percent |
| Not elevated likelihood for ADHD | 44 | 111 | - | - | 155 | 69.82% |
| Sibling diagnosed with ADHD | - | - | 40 | 6 | 46 | 20.72% |
| Parent diagnosed with ADHD | - | - | 5 | 10 | 15 | 6.76% |
| Sibling and parent diagnosed with ADHD | - | - | 5 | 0 | 5 | 2.25% |
| Half-sibling diagnosed with ADHD | - | - | 0 | 1 | 1 | 0.45% |
| Total | 44 | 111 | 50 | 17 | 222 |  |

## Appendix S1: Parent-child Similarity Measure

Previous investigations of the similarity-fit hypothesis have operationalised these effects as conventional interaction terms as in the below example. This approach is ostensibly taken because similarity-fit effects are thought to be moderators of the main effects of parent and/or child neurodevelopmental traits, rather than parent-child trait similarity itself serving as a broader resilience or protective factor.

**Conventional interaction term calculation:**

- - Parent trait autism standardised score * Child trait autism standardised score

We considered using this approach but observed several limitations, most notably that interaction terms do not represent trait similarity per se, but instead capture mutual (or opposing) deviations of children and parents from their respective standardised means of zero. This results in a multiplicative weighting wherein more extreme deviations result in exponentially higher similarity/dissimilarity scores as indexed by the interaction term. Conversely, if a parent and/or child in a given pair scores near the mean of zero, the interaction term for that pair is proportionally downweighted to near-zero. In other words, the highest (and lowest) interaction-based similarity scores are reserved only for pairs in which both parents and children score well above or below the mean. We observed that this approach caused parent-child pairs with very different scores to be treated as substantially more similar than those with very similar scores, due only to the fact that the latter cases were more average-scoring.

Moreover, the bias towards more extreme interaction term scores was directional in our sample, with the highest interaction term scores being seen disproportionately in parent-child pairs where both scored highly on autism and ADHD traits. We aimed to construct a measure that more accurately captures parent-child trait similarity, by computing the absolute difference between standardised parent and child autism and ADHD scores, and then reversing it to attain a measure of absolute similarity. The computation steps are intuitive and can be applied to any pair of dimensional measures for which an index of sample-centred, non-directional similarity is needed. An example of the computation process is given below.

**Absolute similarity score calculation:**

**Step 1:** Compute absolute difference between standardised child and parent scores:

- - AbsDiffZ = abs(Parent trait autism Z-score - Child trait autism Z-score)

**Step 2:** Reverse absolute difference score to derive a sample-centred absolute similarity score, wherein the most dissimilar pair (i.e., highest absolute difference) is assigned an anchoring score of zero, with progressively more similar (i.e., lower absolute difference) pairs scoring more highly on the similarity measure.

- - AbsSimZ = max(AbsDiffZ) - AbsDiffZ

Figure S1 illustrates the distributional differences between conventional interaction terms and our computed similarity measure by illustrating how scores on each index correspond to the difference (or distance) between parent and child scores.

We have created an openly available R Statistics function which allows for the execution of these steps for any two continuous variables within a dataframe, creating an absolute similarity score as used in our analyses. The function carries out various data checks and warns the user about potential issues, allows for the input of auxiliary outcome or other analytic variables to ensure appropriate standardisation for the analytic sample, and can produce plots as seen in Figure S1. The function is currently available as a Github repository at <https://github.com/dlwech/absSim>, and we hope it will be used by other researchers aiming to assess associations between intra-pair similarity on various traits and their outcomes.


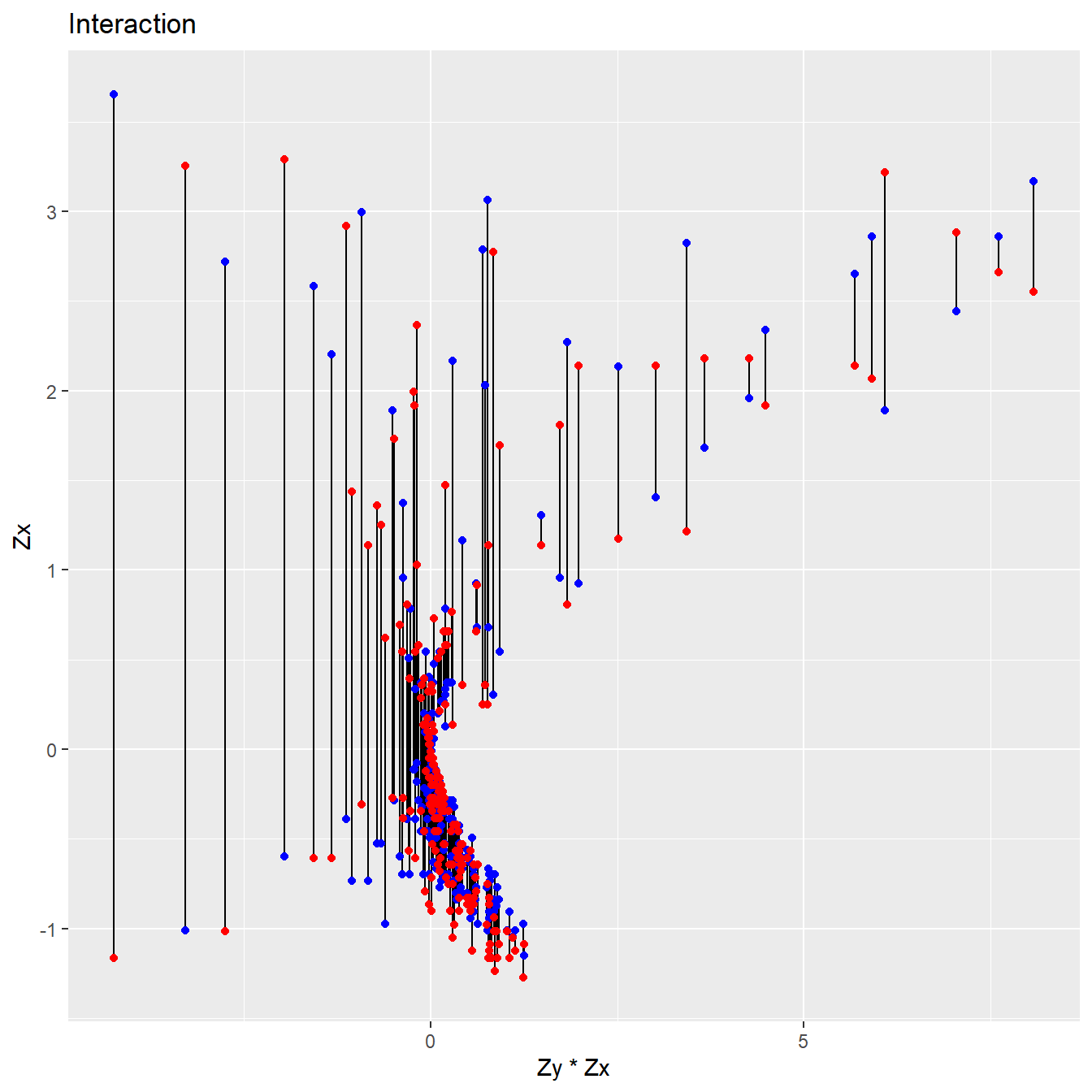

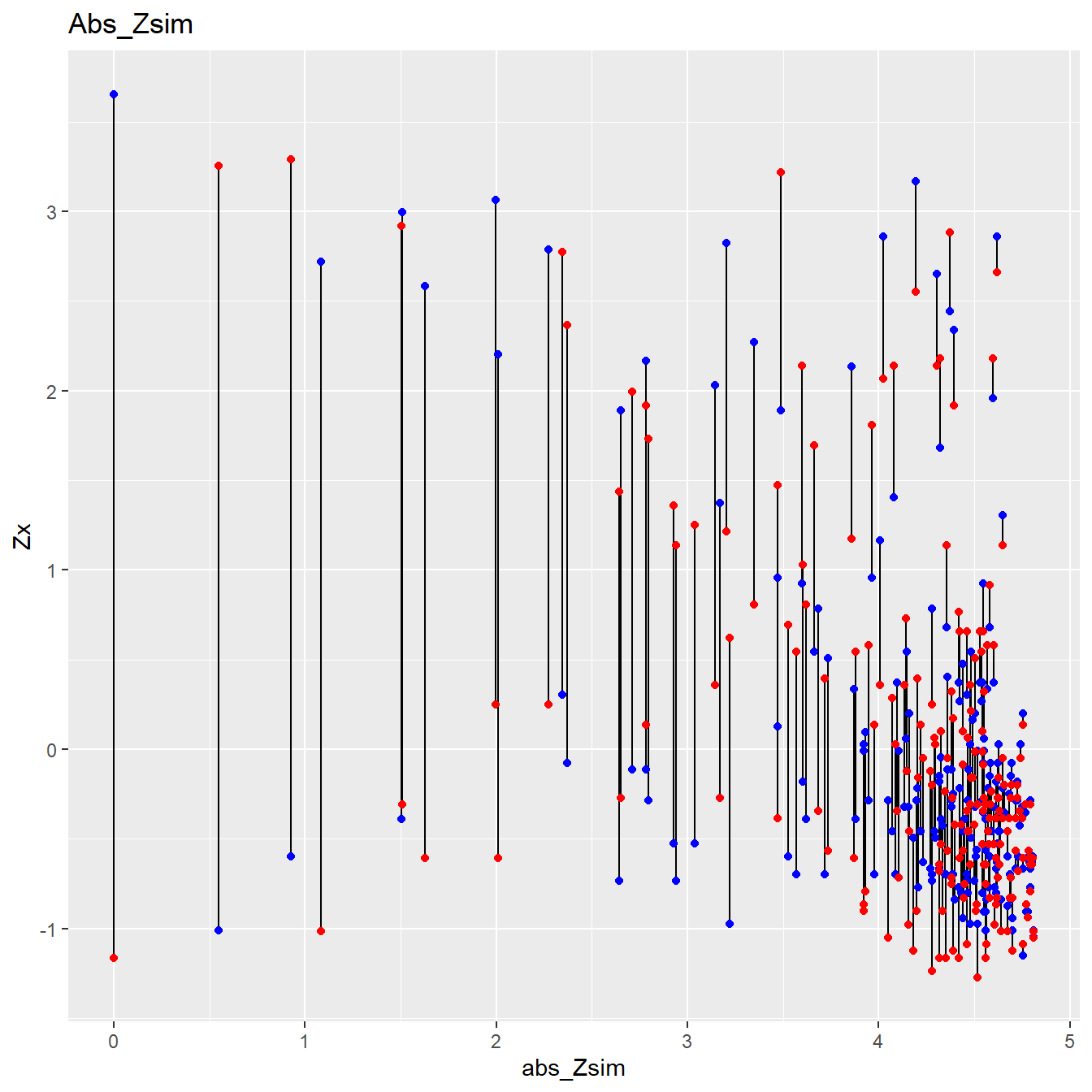

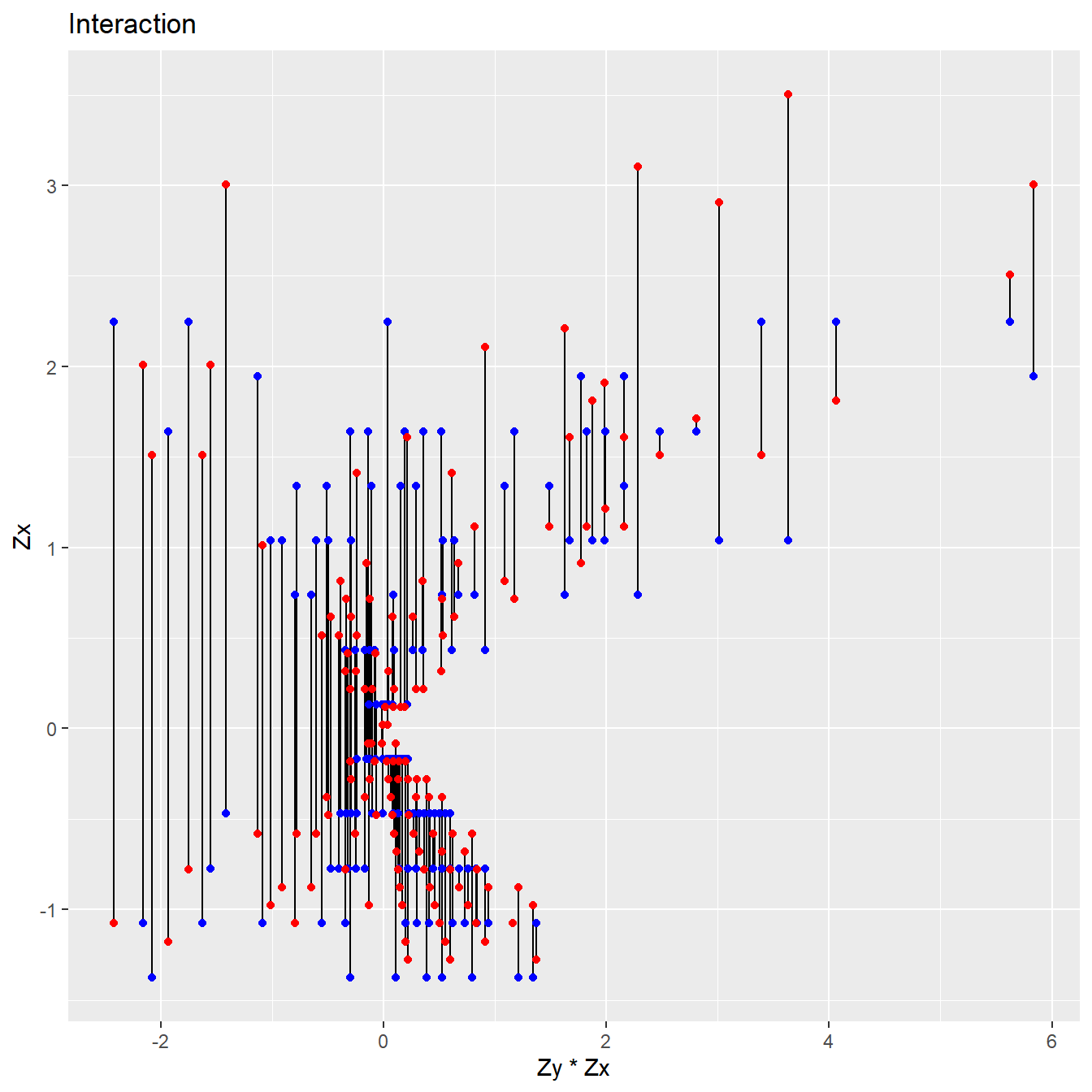

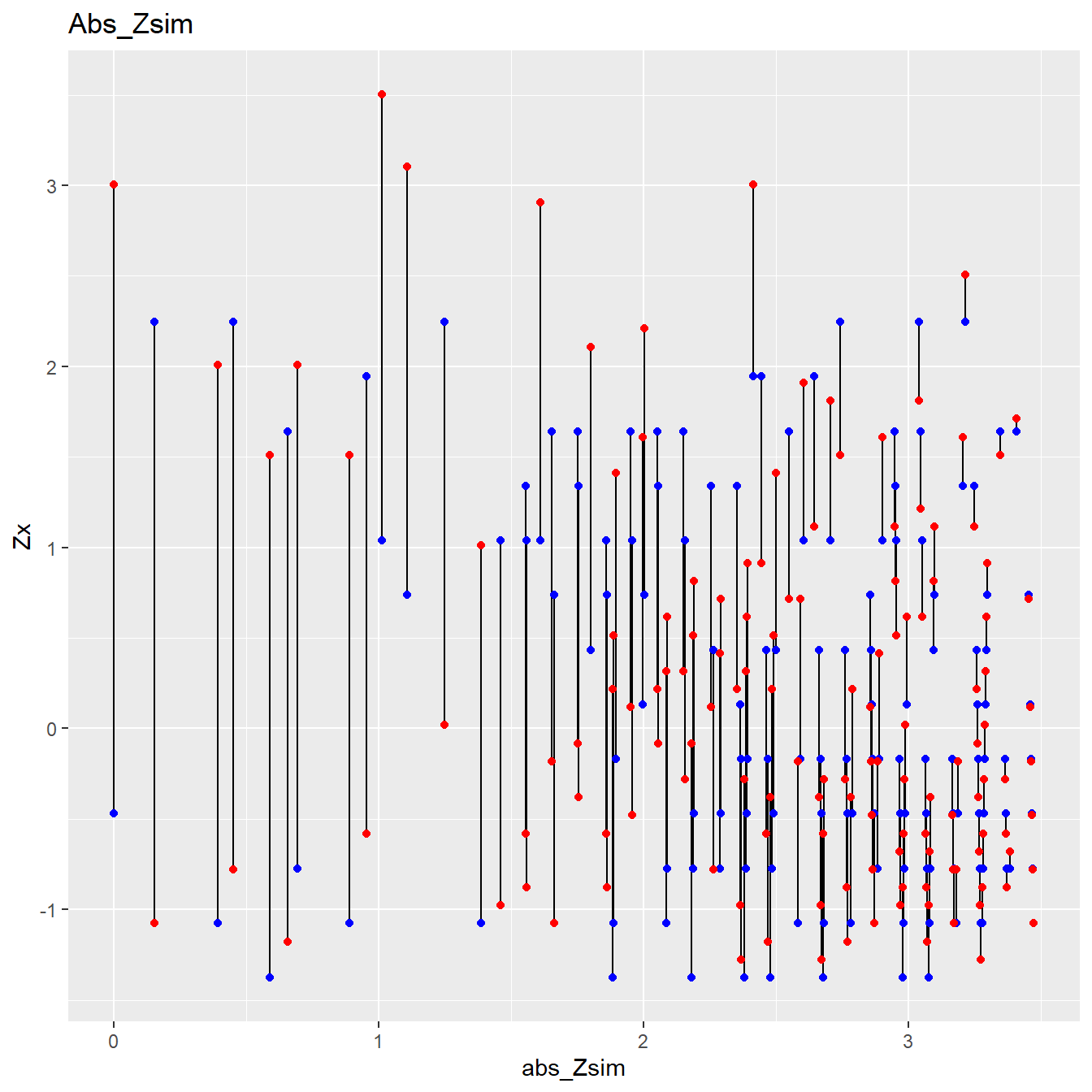

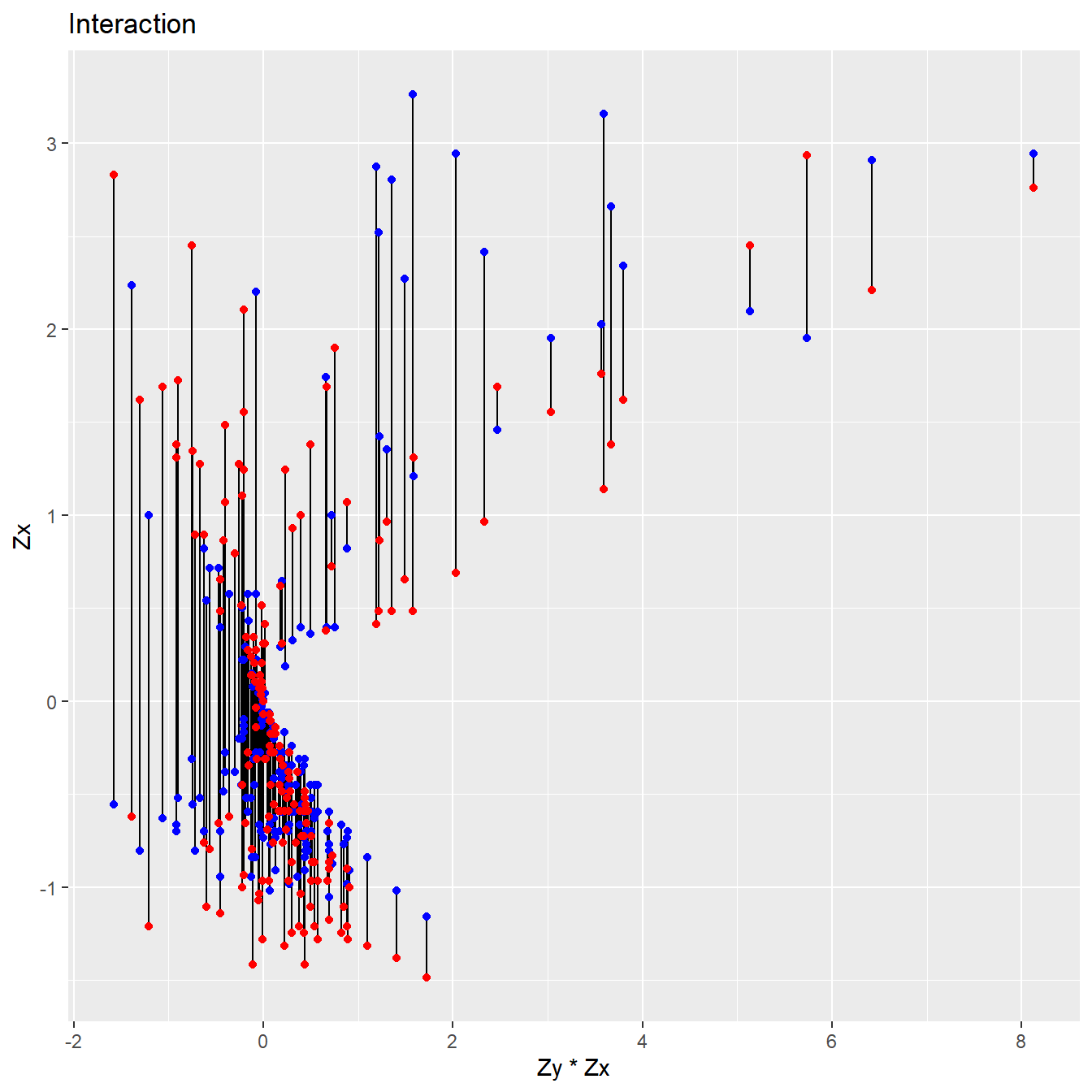

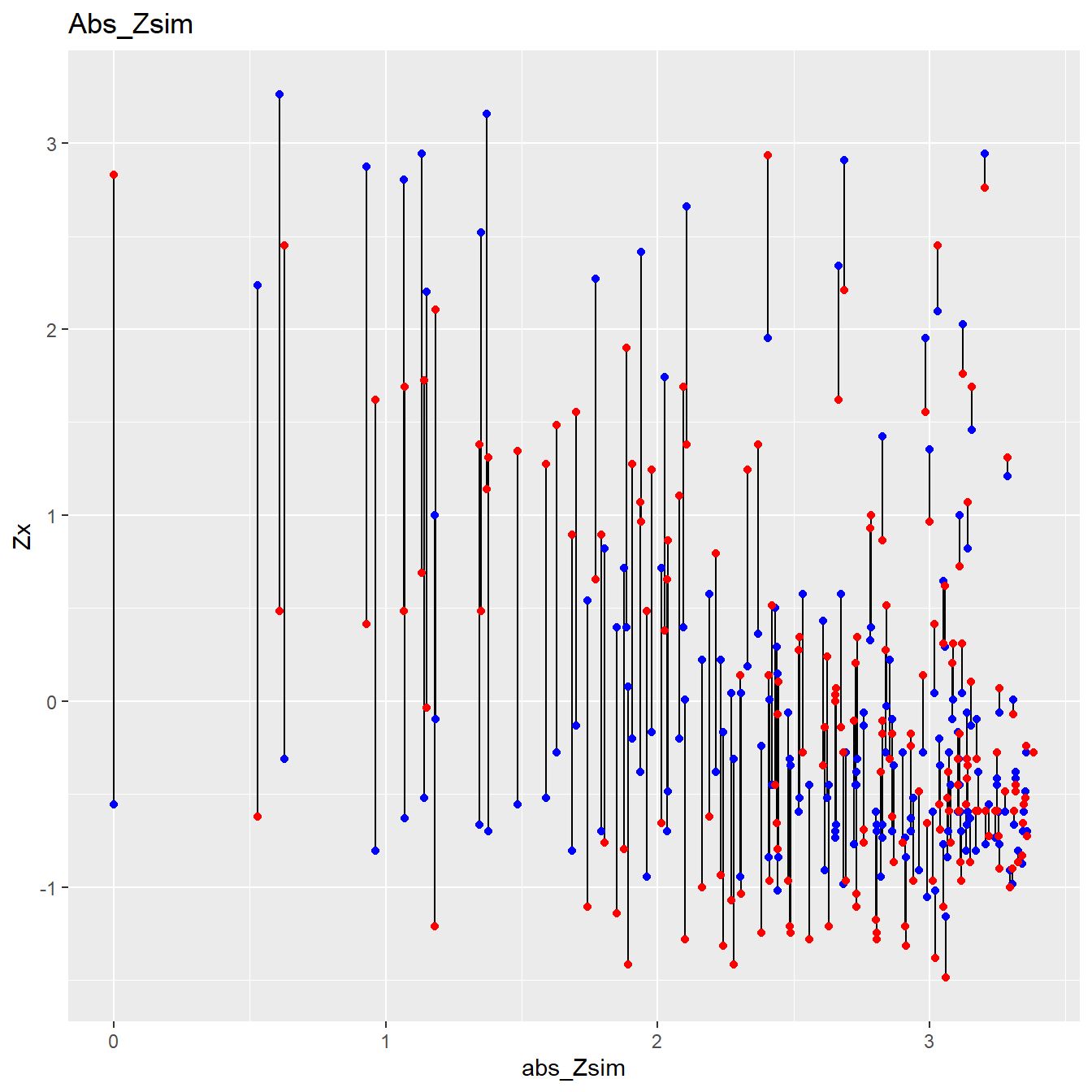

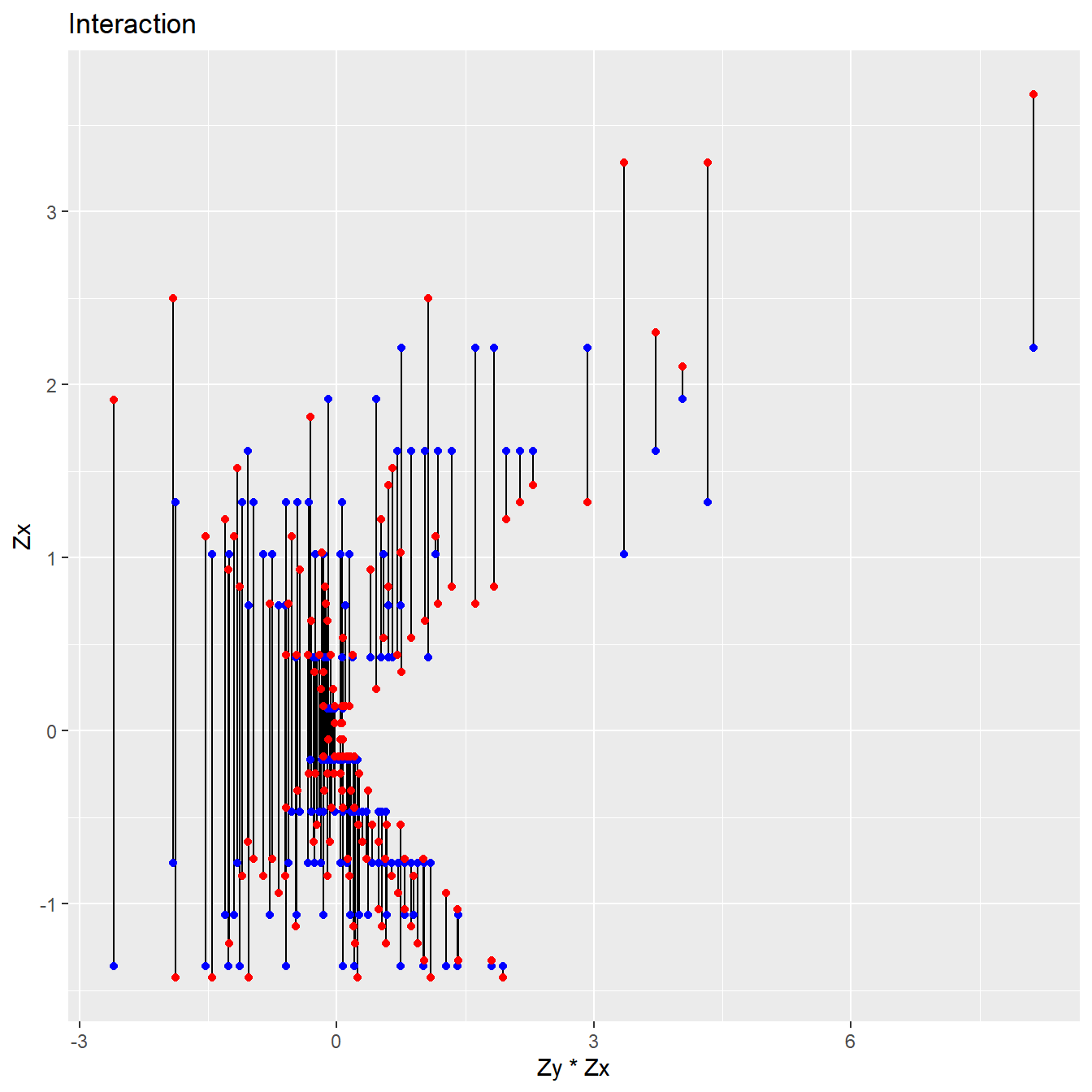

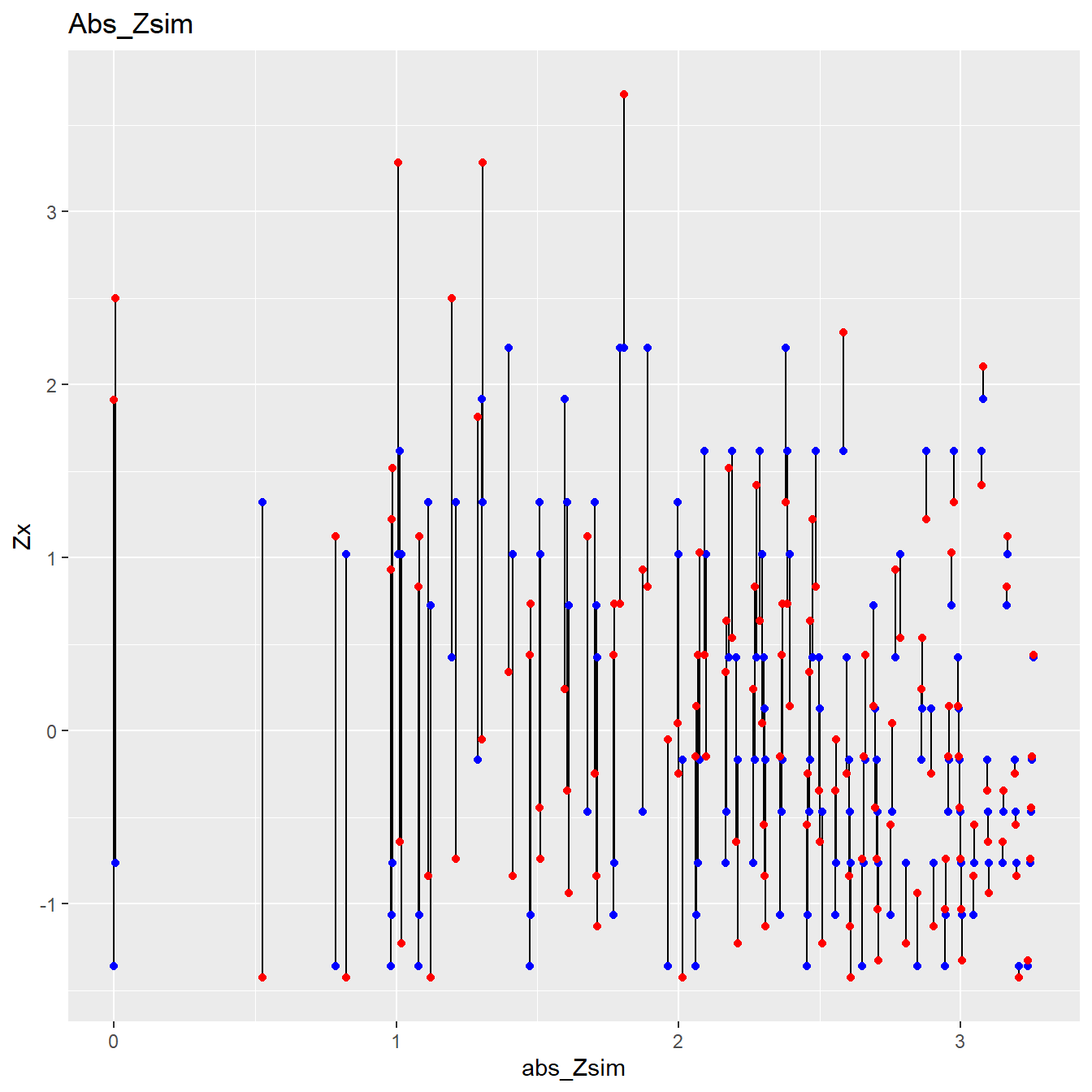


**Figure S1.** Plots comparing interaction terms with absolute similarity measures. Parent (red) and child (blue) standardised trait scores are represented on the y-axis. Each pair’s interaction term (top row) and absolute similarity score (bottom row) is represented on the x-axis. Left to right: Mother-child autism, mother-child ADHD, father-child autism, father-child ADHD.

## Appendix S2: Transformations

Residual diagnostics from regressions using raw variables indicated significant heteroscedasticity and non-normality of errors in both Main Effects and Full models. Most measures were highly skewed, with some evidence of bimodality of child and parent autism and ADHD scores. Accordingly, many bivariate predictor-outcome associations were disproportionately driven by a minority of extreme scores (e.g., very high child SRS scores coinciding with very low Vineland Socialisation scores). To address these issues, we applied Box-Cox power transformations to predictors and outcomes as appropriate using the Forecast R library’s BoxCox.lambda function to derive the best-performing lambda within the standard bounds of -1 and 2 using the Guerrero (1993) method.

We first transformed all autism and ADHD measures given their notable positive skewness and bimodality. Transformations improved the uniformity of bivariate correlations but created 1 to 2 artificial outliers per paired sample on SRS scores, wherein the lowest scoring case(s) on raw measures became extreme negative outliers on transformed measures. As these outliers were artificial (i.e., not reflecting the data-generating process) and the exclusion of outliers on predictor variables does not undermine the statistical and inferential value of regressions (Wilcox, 2022), we opted to exclude these cases after confirming that they were not also outliers on outcome measures. This did not affect the overall pattern of substantive findings, but consistently improved correlations between raw and transformed variables, residual diagnostics, and overall fit as indicated by adjusted R^2^. Any remaining error term non-normality was resolved by transforming outcome measures, which was an acceptable solution for all models except those predicting Vineland Socialisation scores from SRS scores. For these models, adequate fit was achieved using transformed SRS scores and raw Vineland scores.

| **Table S3.** Pearson’s *r* correlations between measures across combined analytic sample. | | | | | | | | | | | | |
| --- | --- | --- | --- | --- | --- | --- | --- | --- | --- | --- | --- | --- |
|  | VABS | CBCL Affective | CBCL Anxiety | SRS | CBCL ADHD | Mother SRS | Mother CAARS | Father SRS | Father CAARS | Sex | Age | Mother Age |
| CBCL Affective Problems | **-.52***** | - | - | - | - | - | - | - | - | - | - | - |
| CBCL Anxiety Problems | **-.52***** | **.65***** | - | - | - | - | - | - | - | - | - | - |
| Child SRS | **-.71***** | **.70***** | **.66***** | - | - | - | - | - | - | - | - | - |
| Child CBCL DSM ADHD | **-.51***** | **.66***** | **.52***** | **.71***** | - | - | - | - | - | - | - | - |
| Mother SRS | **-.24**** | **.34***** | **.29***** | **.44***** | **.38***** | - | - | - | - | - | - | - |
| Mother CAARS | **-.22**** | **.34***** | **.22**** | **.29***** | **.35***** | **.69***** | - | - | - | - | - | - |
| Father SRS | **-.32***** | **.42***** | **.32***** | **.40***** | **.41***** | **.31***** | **.37***** | - | - | - | - | - |
| Father CAARS | **-.17*** | **.36***** | **.26**** | **.32***** | **.31***** | **.34***** | **.32***** | **.69***** | - | - | - | - |
| Child Sex | **.21**** | -.04 | .03 | -.11 | **-.18*** | .03 | -.14 | -.02 | .13 | - | - | - |
| Child Age | -.09 | .02 | .04 | .05 | <.01 | .10 | .09 | .09 | -.01 | -.05 | - | - |
| Mother Age | **.15*** | **-.33***** | **-.20**** | **-.20**** | **-.19*** | **-.18*** | **-.15*** | **-.17*** | **-.22**** | .05 | -.04 | - |
| Father Age | .01 | **-.27***** | -.15 | -.07 | -.11 | -.12 | **-.17*** | **-.24**** | **-.26**** | .02 | -.08 | **.65***** |
| **** p < .001; ** p <.01; * p < .05. Abbreviations: CBCL, Child Behavior Checklist; SRS, Social Responsiveness Scale; CAARS, Conners Adult ADHD Rating Scales; VABS, Vineland Socialisation Dimension Standard Score.* | | | | | | | | | | | | |

## Appendix S3: Covariate Effects

Female child sex predicted higher child social functioning in all autism models, but lower psychological wellbeing in mother-child ADHD models. Higher parent age predicted higher child psychological wellbeing in all models except father-child ADHD MM models. Child age and parent-child data collection interval did not predict any outcome.

| Table S4. Standardised parameter estimates of covariate effects from OLS regressions. | | | | | | | |
| --- | --- | --- | --- | --- | --- | --- | --- |
| Similarity Phenotype | Child Outcome | N | Model | β (p) | | | |
|  |  |  |  | Child Sex | Child Age | Parent Age | Collection Interval |
| Mother-child Autism | Social Functioning | 179 | 1 | **.39 (.002)**** | -.09 (.152) | .05 (.424) | .04 (.552) |
|  |  | 179 | 2 | **.41 (<.001)***** | -.08 (.192) | .04 (.475) | .04 (.492) |
|  | Psychological Wellbeing | 172 | 1 | -.08 (.515) | -.03 (.596) | **.17 (.004)**** | .03 (.627) |
|  |  | 172 | 2 | -.04 (.755) | -.03 (.648) | **.17 (.004)**** | .05 (.428) |
| Mother-child ADHD | Social Functioning | 158 | 1 | .18 (.248) | -.03 (.676) | .01 (.875) | .08 (.302) |
|  |  | 158 | 2 | .17 (.272) | -.01 (.854) | .03 (.717) | .07 (.394) |
|  | Psychological Wellbeing | 182 | 1 | **-.28 (.023)*** | -.07 (.260) | **.18 (.002)**** | .10 (.138) |
|  |  | 182 | 2 | **-.28 (.022)*** | -.06 (.329) | **.19 (.002)**** | .09 (.173) |
| Father-child Autism | Social Functioning | 155 | 1 | **.37 (.003)**** | -.10 (.102) | -.05 (.416) | .03 (.600) |
|  |  | 155 | 2 | **.38 (.002)**** | -.10 (.098) | -.05 (.439) | .03 (.585) |
|  | Psychological Wellbeing | 151 | 1 | -.08 (.493) | -.03 (.656) | **.16 (.010)**** | .03 (.689) |
|  |  | 151 | 2 | -.08 (.490) | -.03 (.665) | **.16 (.010)*** | .03 (.697) |
| Father-child ADHD | Social Functioning | 134 | 1 | .19 (.254) | -.07 (.445) | -.11 (.188) | .06 (.500) |
|  |  | 134 | 2 | .19 (.257) | -.07 (.446) | -.11 (.199) | .06 (.506) |
|  | Psychological Wellbeing | 155 | 1 | -.20 (.146) | -.11 (.139) | **.13 (.045)*** | .08 (.297) |
|  |  | 155 | 2 | -.20 (.149) | -.11 (.139) | **.14 (.046)*** | .08 (.305) |
| **** p < .001; ** p <.01; * p < .05.* | | | | | | | |

| Table S5. Standardised parameter estimates of covariate effects from MM regressions. | | | | | | | |
| --- | --- | --- | --- | --- | --- | --- | --- |
| Similarity Phenotype | Child Outcome | N | Model | β (p) | | | |
|  |  |  |  | Child Sex | Child Age | Parent Age | Collection Interval |
| Mother-child Autism | Social Functioning | 179 | 1 | **.39 (.002)**** | -.08 (.223) | .06 (.339) | .04 (.535) |
|  |  | 179 | 2 | **.43 (<.001)***** | -.07 (.274) | .05 (.449) | .04 (.505) |
|  | Psychological Wellbeing | 172 | 1 | -.06 (.641) | -.02 (.703) | **.19 (.001)**** | .03 (.680) |
|  |  | 172 | 2 | -.03 (.774) | -.02 (.758) | **.17 (.006)**** | .05 (.486) |
| Mother-child ADHD | Social Functioning | 158 | 1 | .20 (.211) | -.03 (.716) | .02 (.807) | .09 (.273) |
|  |  | 158 | 2 | .18 (.251) | -.02 (.847) | .03 (.671) | .08 (.325) |
|  | Psychological Wellbeing | 182 | 1 | **-.30 (.014)*** | -.07 (.310) | **.21 (<.001)***** | .10 (.124) |
|  |  | 182 | 2 | **-.31 (.013)*** | -.06 (.369) | **.21 (<.001)***** | .09 (.150) |
| Father-child Autism | Social Functioning | 155 | 1 | **.40 (.001)**** | -.09 (.160) | -.06 (.333) | .04 (.548) |
|  |  | 155 | 2 | **.42 (<.001)***** | -.09 (.163) | -.05 (.385) | .04 (.517) |
|  | Psychological Wellbeing | 151 | 1 | -.03 (.817) | -.02 (.790) | **.17 (.007)**** | <.01 (.942) |
|  |  | 151 | 2 | -.03 (.805) | -.02 (.802) | **.17 (.008)**** | <.01 (.958) |
| Father-child ADHD | Social Functioning | 134 | 1 | .21 (.233) | -.07 (.478) | -.11 (.201) | .07 (.442) |
|  |  | 134 | 2 | .21 (.242) | -.07 (.482) | -.11 (.204) | .07 (.443) |
|  | Psychological Wellbeing | 155 | 1 | -.21 (.144) | -.11 (.141) | .13 (.059) | .08 (.305) |
|  |  | 155 | 2 | -.21 (.147) | -.11 (.142) | .14 (.062) | .08 (.309) |
| **** p < .001; ** p <.01; * p < .05.* | | | | | | | |
